# Minerals and extremely low birth weight infants

**DOI:** 10.1186/1824-7288-41-S1-A37

**Published:** 2015-09-24

**Authors:** Guglielmo Salvatori, Silvia Foligno

**Affiliations:** 1 Department of Medical and Surgical Neonatology, Children's Hospital Bambino Gesù, IRCSS, Rome,00165, Italy

## 

The 80% of bone mineralisation occurs in the third trimester of pregnancy. The daily fetal requirement is 310 mg of calcium and 170 mg of phosphorus[1]. The bone mineral density (BMD) correlates positively with gestational age, weight and length[2]. At birth, extremely premature infants (ELBWI)canpresent hypocalcaemiabecause of interruption of the maternal calcium supply, high level of calcitoninand immature response to PTH. Early parenteral nutrition solutionisnecessary to maintain normal serum calcium and phosphorus levels. Indeed, the exclusive use of dextrose and calcium gluconate leads to thehypophosphatemia, deleterious to intermediary metabolism, intracellular energy transfer and bone mineral homeostasis[3]. The ELBWI are at risk of Osteopenia of prematurity, also known as neonatal metabolic bone disease (MBD). The pathophysiology includesreduction in organic protein matrix (osteopenia) and/or a reduction in mineral component (osteomalacia). MBD causes rickets, fractures and may affect growth in infancy and childhood. The structural basis of osteopenia is decreased thickness or number of trabeculae and/or decreased thickness of the bone cortex. Osteomalaciais a disorder of the physiological process of mineralisation, when the incorporation of mineral into the organic bone matrix is disturbed, whereas “rickets” describes defective mineralisation of growth plate cartilage and its morphological consequences[4]. The ethiology is multifactorial: placental dysfunction, prolonged (>4 weeks) parenteral nutrition, diureticsand steroids treatment, immobilization and inadequate nutrient intake of calcium, phosphorus, and vitamin D[5]. The main ethiology factor is deficit of mineral substrates, whereas the critical factor is lack of phosphorus[4]. It should be needed an adequate mineral supplementation with parenteral and enteral nutrition, although the difficulties to fortify appropriately the human milk. The dosage recommended of vitamin D is between, 400 IU/d (AAP) and 1000 IU/d (ESPGHAN)[6]. Moreover hypotonia and immobilization are an additional risk factors [7], daily passive exercise canimproves the bone mineral content [8]. MBD could be detected by X-Ray, dual-energy x-ray absorptiometry (DEXA) or quantitative ultrasound. The serumdiagnostic markers are alkaline phosphatases, calcium and phosphateserum levels. Preterm infants with low serum phosphate (<2 mmol/l) are at risk of MBD and levels less than 1.8 mmol/l have been strongly associated with the presence of radiographically evident rickets. Because of preterm has a much lower renal phosphate threshold and this is not bound in the plasma like calcium, the tubular reabsorption is the best guide to calculate itssupplementation[8].
